# Chemical synthesis of unique intermetallic TiFe nanostructures originating from the morphology of oxide precursors†

**DOI:** 10.1039/d1na00251a

**Published:** 2021-07-13

**Authors:** Yasukazu Kobayashi, Heng Yi Teah, Nobuko Hanada

**Affiliations:** Interdisciplinary Research Center for Catalytic Chemistry, National Institute of Advanced Industrial Science and Technology (AIST) 1-1-1 Higashi Tsukuba Ibaraki 305-8565 Japan yasu-kobayashi@aist.go.jp; Waseda Research Institute for Science and Engineering, Waseda University Tokyo 169-8555 Japan; Department of Applied Chemistry, Waseda University 3-4-1 Okubo, Shinjuku-ku Tokyo 169-8555 Japan

## Abstract

In this study, intermetallic TiFe nanostructures were chemically prepared from Ti–Fe oxide precursors using a CaH_2_ reducing agent in molten LiCl at as low as 600 °C. The used precursor was spherical oxide nanoparticles or commercial FeTiO_3_ bulk powder. After the reduction treatment, the former precursor was changed to an aggregation of TiFe nanoparticles with a particle size of 44–46 nm. Surprisingly, the latter precursor was reduced to a layered morphology composed of TiFe nanoparticles with a particle size of 47–65 nm. An intermetallic compound with a unique layered morphology was found for the first time, and the layered morphology could have originated from the morphology of the FeTiO_3_ precursor in which the Fe^2+^ and Ti^4+^ ions occupied alternating layers perpendicular to the trigonal *c*-axis. The precursor originated morphology was enabled by the proposed low reduction temperature method, and the environment-friendliness of the proposed method was finally evaluated using life-cycle assessment (LCA).

## Abstract 2

TiFe nanostructures where prepared at temperatures as low as 600 °C with a Ti–Fe precursor and a CaH_2_ reducing agent in molten LiCl. For the first time an intermetallic compound with a unique layered morphology was found which could have originated from the FeTiO_3_ precursor.

## Graphical abstract

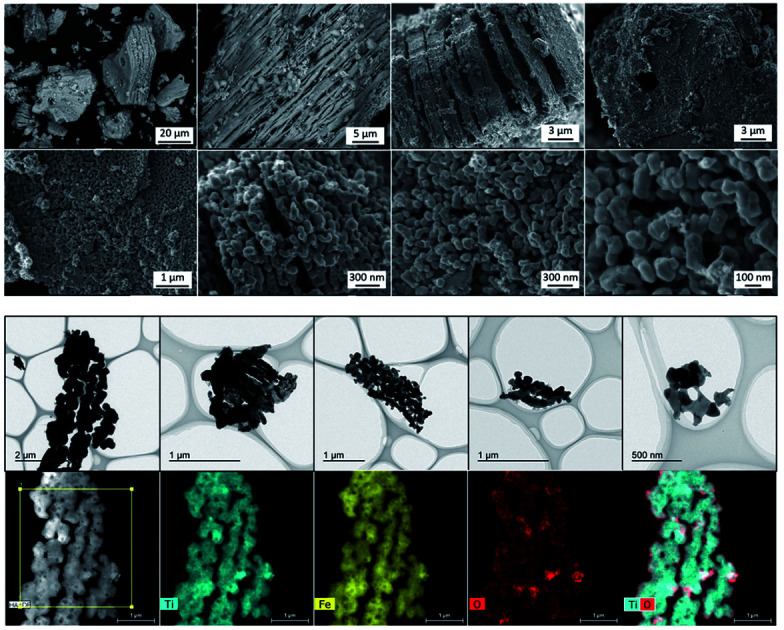

## Introduction

Intermetallic TiFe is one of the most attractive hydrogen storage materials due to its low cost, non-toxicity, and highly reversible hydrogen-absorbing/releasing capacity (up to 1.9 wt%).^1–4^ In traditional methods, TiFe alloys are physically prepared from pure titanium and iron metals as starting materials, where they are melted at a high temperature of ∼2000 °C under an inert atmosphere for well-mixing.^5,6^ Since producing pure titanium from oxide ores, such as FeTiO_3_ and TiO_2_, is an extremely energy-consuming process, chemical approaches, such as electrochemical methods and combustion synthesis methods, have been recently proposed and experimented to directly prepare intermetallic TiFe from titanium oxides at milder conditions. Regarding the electrochemical methods, intermetallic TiFe alloys have been directly prepared from the titanium sources of FeTiO_3_ ilmenites^7–12^ and TiO_2_ (ref. 13) at 900–950 °C in molten CaCl_2_ with a voltage supply of 3.0–3.2 V to reduce the oxides. To further decrease the reduction temperature, Zhou *et al.* used a mixed molten salt of CaCl_2_–NaCl as an electrolyte and finally successfully prepared ferrotitanium powders from ilmenite at the lowest temperature of 700 °C.^14–16^ A small amount of CaO was added to the molten salt to improve the ilmenite reduction rate, and a pure TiFe powder was obtained at 700 °C with a 3.2 V supply for 15 hours, whereas some impurity phases of FeTi_2_ and CaTiO_3_ were observed in the 600 °C electrolysis due to the incomplete ilmenite reduction.^17^ As for the combustion synthesis methods, Ca chips or Mg powder was used as a reducing agent and heat source to prepare TiFe powder from Fe and TiO_2_.^18,19^ A mixture of Fe, TiO_2_, and Ca or Mg was heated until combustion occurred following the reduction of TiO_2_, and the temperature reached ∼1400 °C. After washing with acetic acid, HNO_3_, or HCl (solution) to remove the calcium/magnesium species, pure TiFe powders were successfully obtained. Thus, several chemical methods have succeeded in directly preparing intermetallic TiFe using titanium compounds as a titanium source. Thus, from the viewpoint of saving the environment and limiting climate change, chemical methods are a prominent approach for preparing intermetallic TiFe, and it is ardently aspired to further develop those operated at milder conditions.

In this study, we developed a new chemical method to prepare intermetallic TiFe nanostructures. Titanium oxide (FeTiO_3_ or TiO_2_) was used as titanium sources, and they were reduced using CaH_2_ as a reducing agent in molten LiCl at 600 °C, which is the lowest reduction temperature in comparison with previous reports. Interestingly, the morphologies of the obtained TiFe had an origin to the Ti–Fe oxide precursors, and intermetallic TiFe with a layered morphology was observed in a sample obtained from the layered FeTiO_3_ precursor. Alloys with unique layer morphologies have never been reported before, as far as we know, and they can only be realised by a low reduction temperature through which a rate of grain growth of TiFe is slow. Besides, in comparison with previous chemical approaches, our method required a simple preparation facility with less energy demand. Thus, the proposed chemical method is a highly promising method for scalable applications. To quantitatively evaluate the environmental performance of the prospective TiFe production, a process-based life cycle assessment (LCA) was conducted.

## Experimental

### Preparation of the intermetallic TiFe nanostructures

The intermetallic TiFe nanostructures were prepared using two different titanium sources of bulk FeTiO_3_ powder and TiO_2_ powder composed of nanoparticles. CaH_2_ was used in molten LiCl to reduce the oxides at 600 °C.^20–27^ For the FeTiO_3_ method, commercial FeTiO_3_ powder (Kojundo Chemical Laboratory Co., Ltd.) was mixed in air with CaH_2_ (JUNSEI Chem. Co. Ltd.) and LiCl (Wako Pure Chem. Corp.) in a mortar with a weight ratio of FeTiO_3_/CaH_2_/LiCl = 2/6/3. The mixed powder was then loaded in a stainless-steel reactor and heated at 600 °C for 2 hours under argon gas flow. Finally, the reduced precursor was crushed in a mortar and rinsed using an 0.1 M NH_4_Cl aqueous solution and distilled water to obtain the final product, named TiFe(RDT-FTO). Rinsing treatments were conducted to remove any possible impurity species related to CaH_2_ and LiCl. For the TiO_2_ method, Fe(NO_3_)_2_·6H_2_O (Wako Pure Chem. Corp.) was firstly dissolved in distilled water, and after well-mixing the solution, TiO_2_ nanoparticles (50 nm, Degussa P25, Evonik Industries AG) were suspended into the solution with a stoichiometric molar ratio of Fe/Ti = 1/1. While stirring the suspension, it was kept at a temperature of 110 °C overnight. The dried powder was then calcined at 500 °C in air for 2 hours to obtain the oxide precursor, named TiFe(Pre-TO). Next, the precursor was mixed with CaH_2_ and LiCl in a mortar and treated similarly to the FeTiO_3_ method described above to obtain the final product, named TiFe(RDT-TO).

### Characterisation of the prepared TiFe nanostructures

The crystal structure was examined using X-ray diffraction (XRD, SmartLab (3 kW), Rigaku) with CuKα radiation at 40 kV and 45 mA. The porosity was investigated using N_2_ adsorption/desorption at −196 °C (BELLSORP mini-II, Microtrac-BEL). The sample was pre-treated at 200 °C for 30 min under vacuum before the measurement. The pore size distribution was analysed from the measured isotherms using the Barrett–Joyner–Halenda method. The morphology was observed using a scanning electron microscope (SEM, JSM-7800F, JEOL Ltd.) and a (scanning) transmission electron microscope ((S)TEM, Tecnai Osiris, FEI) with energy dispersive X-ray spectrometry (EDS) for the elemental analysis of the final obtained sample.

### Environmental evaluation of the prospective TiFe production

The environmental performance of the TiFe production using the proposed method was evaluated using a process-based LCA.^28^ The goal was to screen for any potential environmental hotspots from the materials and consumed energy in the whole processes. The cradle-to-gate system boundary is illustrated hereinafter. A process inventory was first conducted on the experiment. Then, an expected value for the prospective production was estimated based on expert judgement. The inventory for the background processes was supplemented using an academic reputed database, the Ecoinvent v3.6.^29^ Two environmental indicators, global warming potential (GWP) and cumulative energy demand (CED), were selected as TiFe is considered a potential low-carbon technology. The GWP and CED were characterised following the openLCA LCIA methods v2.0.4,^30^ and the model was constructed on an openLCA platform.^31^

## Results and discussion

### FeTiO_3_ route to prepare TiFe with a layered morphology

**Fig. 1:**
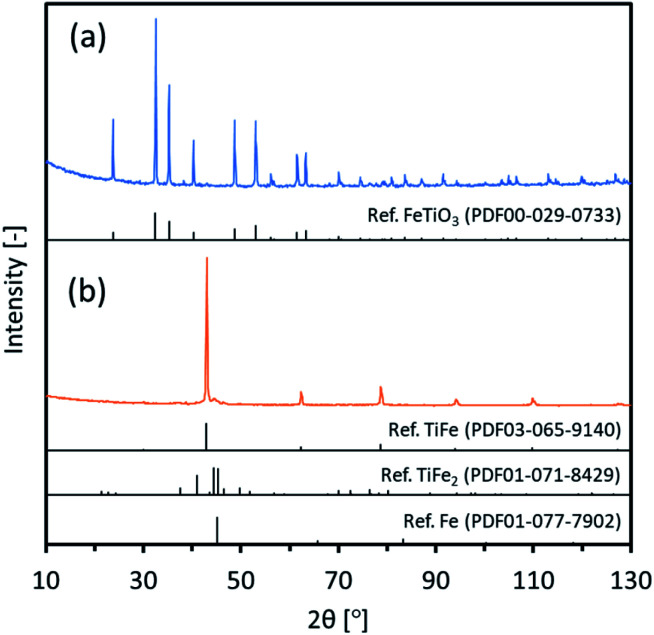
XRD patterns of (a) the commercial FeTiO_3_ and (b) TiFe(RDT-FTO) with possible references.

Intermetallic TiFe nanostructures were prepared using two different methods: FeTiO_3_ and TiO_2_. For the FeTiO_3_ method, commercial FeTiO_3_ powder was directly used as an oxide precursor. Fig. 1(a) shows XRD patterns of commercial FeTiO_3_. The observed peaks were perfectly assigned to a FeTiO_3_ phase. Also, SEM images of commercial FeTiO_3_ are shown in Fig. S1.† It looks to be composed of a micro-sized large mass with good crystallinity. Circle marks were seen on some surfaces, and it was considered that they were mechanically made when the as-prepared sample was crushed into pieces.

Next, the FeTiO_3_ precursor was reduced in a CaH_2_–LiCl mixture and then rinsed to remove any impurity species, and the procedures described in the experimental section were then performed. Fig. 1(b) shows XRD patterns of the final TiFe(RDT-FTO) sample. The peaks assigned to the intermetallic TiFe phase were majorly observed with the main peak of (1 1 0) facet at 43°. The very small peaks observed next to the main peak around 45° could be ascribed to the impurity phases, such as TiFe_2_ and Fe. Rietveld analysis was then carried out on the XRD data with an expectation that it might yield useful data with regard to the state of the reduced powder. Fig. S2† shows the result of Rietveld refinement. Three compounds of TiFe, Fe and TiFe_2_ were identified with a weight percentage of 82.0, 2.9 and 15.1, respectively. The result confirmed that the impurity species were negligible enough in comparison with TiFe. Thus, intermetallic TiFe with less impurity species was successfully prepared from FeTiO_3_ with a mixture of LiCl and CaH_2_ at 600 °C. According to our previous works, it was clarified that LiCl was melted to be in a form of molten salt at 600 °C. In the molten LiCl, CaH_2_ acted as a superior reducing agent to reduce very stable oxides, such as Al_2_O_3_, SiO_2_, Y_2_O_3_, La_2_O_3_, ZrO_2_ and so on.^20–27^ Therefore, it was considered that FeTiO_3_ was reduced by CaH_2_ in molten LiCl to form the intermetallic TiFe directly as below.FeTiO_3_ + 3CaH_2_ → TiFe + 3CaO + 3H_2_

**Fig. 2:**
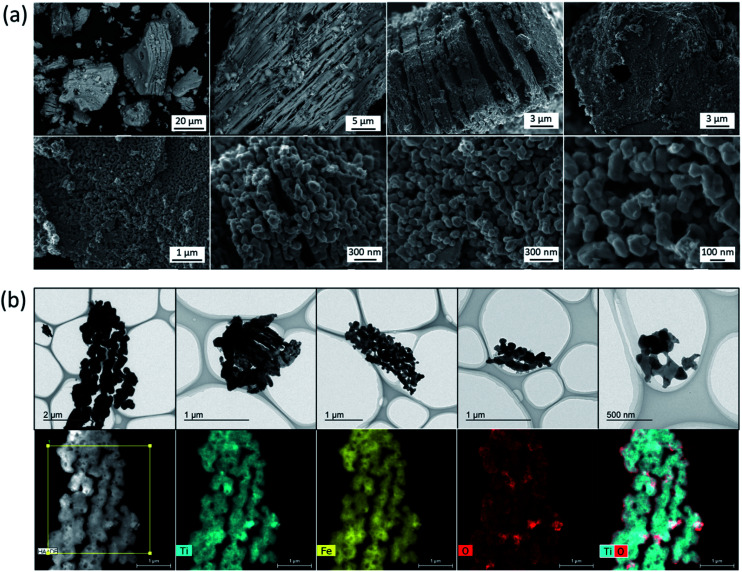
(a) SEM images and (b) TEM images with the elemental mappings of Ti, Fe, O and Ti–O for TiFe(RDT-FTO).

The calculated crystallite size of the TiFe phase from the main peak using the Scherrer equation at 43° was 46.6 nm (Table 1), suggesting a formation of nano-sized TiFe. Fig. 2(a) shows SEM images of TiFe(RDT-FTO). Interestingly, a layered morphology can be clearly seen in a micron size range. Some circle marks were observed on the surface, and they could have originated from the used commercial FeTiO_3_ precursor because the same marks were observed on its surface (Fig. S1†). In the magnified images, the layers seemed to be composed of small nanoparticles with a relatively good size distribution of ∼100 nm. Fig. S3† shows the results of the TiFe(RDT-FTO) elemental analysis by SEM-EDS. Molar ratios of detected elements were summarized on Table S1.† From the elemental mappings in Fig. S3(a),† the elemental distributions of Ti and Fe were fairly good. Also, the elemental analysis corresponding to the layered morphology (Fig. S3(b)†) indicated that the main components were Ti and Fe, and their molar ratio was 46.5/49.3, which is close to the stoichiometric ratio (1) of the intermetallic TiFe phase. Fig. 2(b) shows TEM images with the elemental mappings of Ti, Fe, O and Ti–O for TiFe(RDT-FTO). Layered morphology was slightly observed in the TEM images. Because the sample was observed after sonicated in ethanol in order to distribute the sample particles on the TEM grid, it was speculated that most layered morphology could be destroyed or exfoliated in the sonication treatment, indicating the layers might be connected with each other by a weak interaction. Elemental analysis was conducted in a layered morphology shown in Fig. 2(b). An obtained EDS spectrum was given in Fig. S4.† A detected molar ratio of Ti/Fe was 46.9/48.4 (Table 1). These results also confirmed that the layered morphology observed in TiFe(RDT-FTO) was mainly composed of Ti and Fe. Taking the results of XRD, SEM-EDS and TEM-EDS into consideration, the unique layered compounds were identical to intermetallic TiFe. Apart from the main components of Ti and Fe, some impurity elements (O, Al, Si) were detected using an EDS analysis (Table S1†). The Al and Si amounts were negligible, and they could have been incorporated from the used reactor in the reduction process. However, it was found that the detected O amount was relatively large. Since Ti is highly sensitive to air and can be easily oxidised, it was suggested that the surface of the obtained sample could have been oxidised, thus forming titanium oxides. High oxygen concentration on surface, which was observed in the elemental mappings of Ti–O (Fig. 2(b)), also indicated the formation of oxide films on the surface of TiFe(RDT-FTO).

**Table 1:** BET surface area (SA), pore volume (*V*_p_), particle size calculated using the nitrogen adsorption and XRD measurements and molar ratios of Ti, Fe and O measured by SEM-/TEM-EDS

Sample	SA [m^2^ g^−1^]	*V*_p_ [cm^3^ g^−1^]	Particle size [nm]		Molar ratio of main constituent elements [mol%]				
			N_2_ ads^a^	XRD^b^	Method^c^	Ti	Fe	O	Average of Ti/Fe
TiFe(RDT-FTO)	13.9	0.022	65.2	46.6	SEM1	46.5	49.3	4.2	1.00/1.05
					SEM2	43.2	45.6	11.2	
					TEM	46.9	48.4	4.7	
TiFe(RDT-TO)	20.8	0.039	43.5	45.7	SEM1	43.9	47.9	8.2	1.00/1.12
					SEM2	43.2	49.6	7.2	

a It was assumed that the samples were composed of non-porous spheres with a density of 6.64 g cm^−3^ for FeTi.

b Calculated using the Scherrer equation with peaks observed at 43.0° for FeTi.

c Element ratios were measured by SEM-EDS at 2 different positions for TiFe(RDT-FTO) and TiFe(RDT-TO), and TEM-EDS only for TiFe(RDT-TO).

### TiO_2_ route to prepare TiFe nanoparticles

**Fig. 3:**
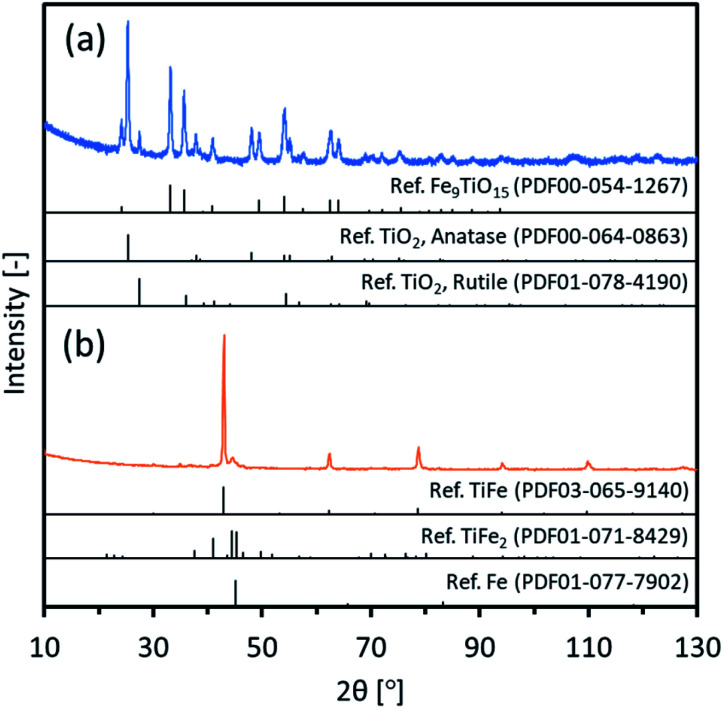
XRD patterns of (a) TiFe(Pre-TO) and (b) TiFe(RDT-TO) with possible references.

In the TiO_2_ method, the oxide precursor, TiFe(Pre-TO), was prepared by loading Fe(NO_3_)_2_ onto spherical TiO_2_ nanoparticles (50 nm) and subsequently heating them to form an oxide. Fig. 3(a) indicates XRD patterns of TiFe(Pre-TO). The observed peaks were assigned to three different oxides: Fe_9_TiO_15_, anatase-type TiO_2_ and rutile-type TiO_2_. Since the used TiO_2_ nanoparticles were originally a mixture of anatase and rutile phases, the detected TiO_2_ phases in TiFe(Pre-TO) could be of unreacted original phases. For the detected Fe_9_TiO_15_, it could mainly exist on the TiFe(Pre-TO) surface because the Fe species was loaded on the TiO_2_ nanoparticles surface. Thus, it was considered that TiFe(Pre-TO) had a core–shell structure, which was probably formed with the core and shell of the unreacted TiO_2_ and reacted Fe_9_TiO_15_, respectively. Fig. S5† shows SEM images of TiFe(Pre-TO). As expected from the used TiO_2_ nanoparticles (50 nm), TiFe(Pre-TO) looked to be composed of nanoparticles with a good size distribution of <100 nm. Next, TiFe(Pre-TO) was reduced and rinsed in a similar manner to that of the FeTiO_3_ method. Fig. 3(b) shows XRD patterns of TiFe(RDT-TO). The observed peaks were mostly identified to an intermetallic TiFe phase. Other than the TiFe peaks, negligible peaks were observed around 45°, and they could be of the impurity phases, such as TiFe_2_ and Fe, similar to the FeTiO_3_ method. Rietveld analysis was then carried out on the XRD data of TiFe(RDT-TO). Fig. S6† shows the result of Rietveld refinement. Three compounds of TiFe, Fe and TiFe_2_ were identified with a weight percentage of 73.0, 6.11 and 20.9, respectively. The result confirmed that the impurity species were negligible enough in comparison with TiFe. Since FeTiO_3_ was totally reduced by CaH_2_ in molten LiCl at 600 °C in the FeTiO_3_ method, it was considered in the TiO_2_ method that the oxide precursors of Fe_9_TiO_15_ and TiO_2_ were similarly reduced in molten LiCl–CaH_2_ system to form intermetallic TiFe as follows.Fe_9_TiO_15_ + 8TiO_2_ + 31CaH_2_ → 9TiFe + 31CaO + 31H_2_

**Fig. 4:**
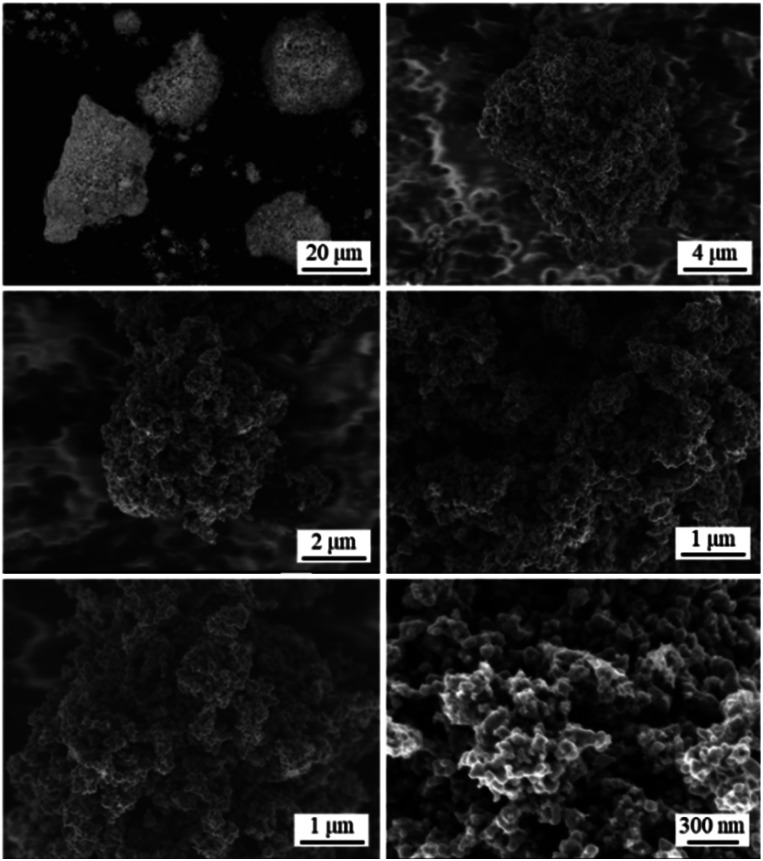
SEM images for TiFe(RDT-TO).

Fig. 4 shows SEM images of TiFe(RDT-TO). In a micron size range, TiFe(RDT-TO) looked like an irregularly-shaped mass without any specific morphology. However, in the magnified images, it was obvious that the mass was an aggregation of nanoparticles ∼100 nm. Fig. S7† shows the results of the SEM-EDS analysis for TiFe(RDT-TO). From the elemental mappings of Ti and Fe, slight difference was observed in their distribution on TiFe(RDT-TO). Because some impurity species of Fe (6.11 wt%) and TiFe_2_ (20.9 wt%) were identified by Rietveld analysis, the difference could result from the influence of the species. The detected molar ratios of Ti/Fe/O and all elements at different two positions are shown on Tables 1 and S1,† respectively. Average Ti/Fe molar ratio of 1.00/1.12 is a good match to the stoichiometric ratio in the intermetallic TiFe phase. In addition to Al and Si, which were detected in the case of the FeTiO_3_ method, a small amount of Ca was also detected in the TiO_2_ method. Possible Ca species were left in the sample, such as CaH_2_, CaO, CaCl_2_, *etc.* However, they could be removed using an NH_4_Cl solution rinsing treatment. The oxygen amount was relatively large, similar to the FeTiO_3_ method, which probably indicates that the TiFe(RDT-TO) surface could have been passivated by a thin oxide layer of a few nanometers that was not detected by the XRD measurements. Overall, intermetallic TiFe nanoparticles with high crystallinity were successfully obtained using the TiO_2_ method.

### Morphology formation mechanism for the TiFe nanostructures

To examine the porosity of the obtained samples, nitrogen adsorption/desorption experiments were performed. Fig. S8† shows the measured nitrogen adsorption/desorption isotherms and the corresponding pore size distributions for TiFe(RDT-FTO) and TiFe(RDT-TO). The obtained physical values were summarised in Table 1. Hystereses were hardly observed between the adsorption and desorption isotherms for both samples, and the pore volumes were so small. The measured BET surface areas were 13.9 m^2^ g^−1^ and 20.8 m^2^ g^−1^, respectively. The average particle sizes estimated from the BET surface areas were 65.2 nm and 43.5 nm, respectively. These values were nearly the same as the crystallite sizes of 46.6 nm and 45.7 nm, which were estimated by XRD measurements. Besides, a lot of nanoparticles (<100 nm), which were component elements for the micro-sized morphologies, were observed in both the SEM images.

**Fig. 5:**
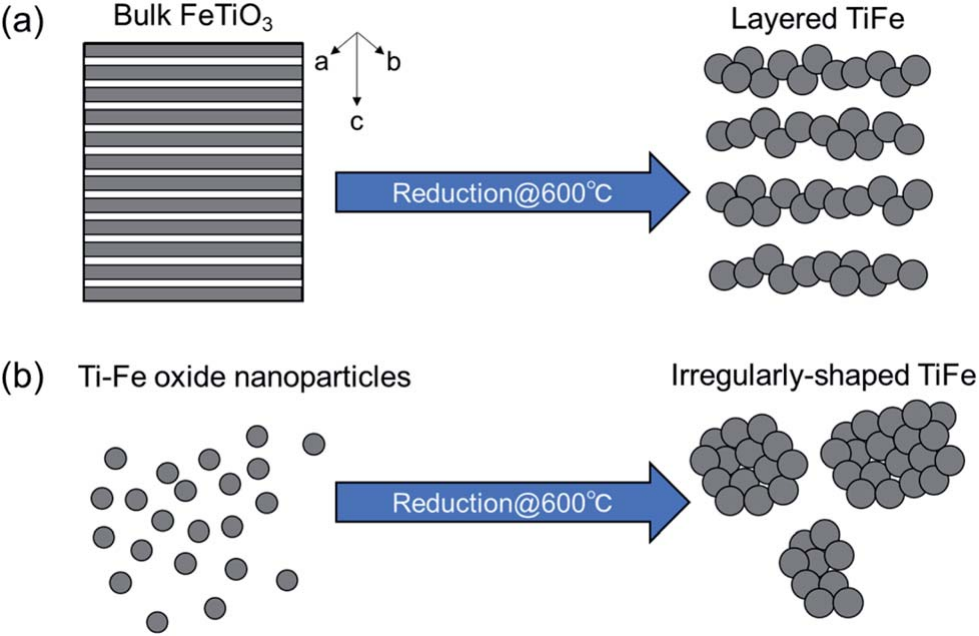
Possible morphology formation for (a) TiFe(RDT-FTO) and (b) TiFe(RDT-TO).

Intermetallic TiFe nanostructures were prepared using two different manners: FeTiO_3_ and TiO_2_. Although the crystal structures (TiFe) and component elements (nanoparticles) were the same in both TiFe(RDT-FTO) and TiFe(RDT-TO), their morphologies were totally different from each other. A unique layered morphology was obtained only *via* the FeTiO_3_ method, in which highly crystalised FeTiO_3_ powder was directly reduced to produce intermetallic TiFe. The FeTiO_3_ structure consisted of alternating layers of Fe and Ti perpendicular to the hexagonal *c*-axis with intervening oxygen layers.^32^ If only the oxygen layers were taken out form the FeTiO_3_ structure using a mild reduction process, the reduced TiFe alloy could have kept the original layered structure of FeTiO_3_ with a subsequent slow alloying process. In the previous studies, TiFe was prepared from TiFeO_3_ at 700–950 °C.^7–16^ This temperature range is quite high enough to promote alloy growth, and the obtained TiFe alloys actually had irregular morphologies with a large micron size. However, our reduction temperature was 600 °C, so the alloying rate can be slower than that of previous studies after the FeTiO_3_ reduction. Thus, a low-temperature reduction can allow the reduction of FeTiO_3_ while maintaining the layered structure of FeTiO_3_ to some extent (Fig. 5).

### Environmental evaluation of the prospective TiFe production

**Fig. 6:**
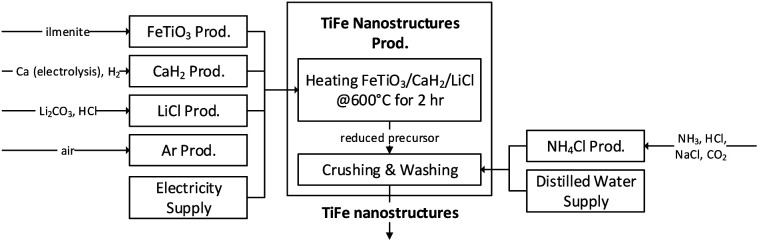
A cradle-to-gate system boundary of the LCA of the TiFe nanostructures production with the proposed chemical synthesis method.

Intermetallic TiFe nanostructures were successfully and directly prepared from oxide precursors, FeTiO_3_ and TiO_2_, using the proposed chemical method. The cradle-to-gate system boundary was illustrated, as shown in Fig. 6. In this method, a reduction temperature of 600 °C was used, and this is the lowest used temperature among the chemical methods used for preparing titanium to date. Also, the proposed method required simpler facilities without a continuous electric supply, which is in contrast to the previously reported electrochemical methods. Thus, our proposed chemical method is considered a prominent approach. An LCA was further conducted to ensure the environmental performance of the proposed method for prospective production, and it was performed based on the expected inventory instead of the experimental inventory. The excessive use of materials and inefficient use of energy are common in laboratory studies. For example, the furnace was only partially filled with a small number of samples, but the heating energy was about the same per batch of reaction. Therefore, decreases in most of the requirements would resemble a more realistic production. Table 2 shows the inventories of the FeTiO_3_ method and the rationale of estimation.

**Table 2:** Life cycle inventory of the experimental data (experiment) and expected data (expectation) for the prospective production of 1 kg TiFe

Input	Unit	Experiment	Expectation	Rationale
**P1 heating FeTiO_3_/CaH_2_/LiCl**				
FeTiO_3_	kg	1.46	1.46	—
CaH_2_	kg	4.39	0.80	Theoretical minimum requirement is FeTiO_3_ : CaH_2_ : LiCl = 1 : 0.55 : 0.27 in weight ratio based on TiFeO_3_ + 2CaH_2_ → TiFe + 2CaO + H_2_O + H_2_
LiCl	kg	2.19	0.39	
Argon	kg	0.41	0.01	Minimum requirement of inert gas to fully fill the reactor
Electricity	kW h	3571	26	Batch reaction with maximum volume capacity of sample to improve the energy efficiency based on a 500 W electric furnace (http://www.asahi-rika.co.jp/tube/arf_kc.html)

**P2 crushing & washing**				
NH_4_Cl	kg	36	0.04	Assume volume ratio of cleaning agents: sample = 50 : 1 considering efficient process and reusability of cleaning agents
Distilled water	kg	7143	15	

**Fig. 7:**
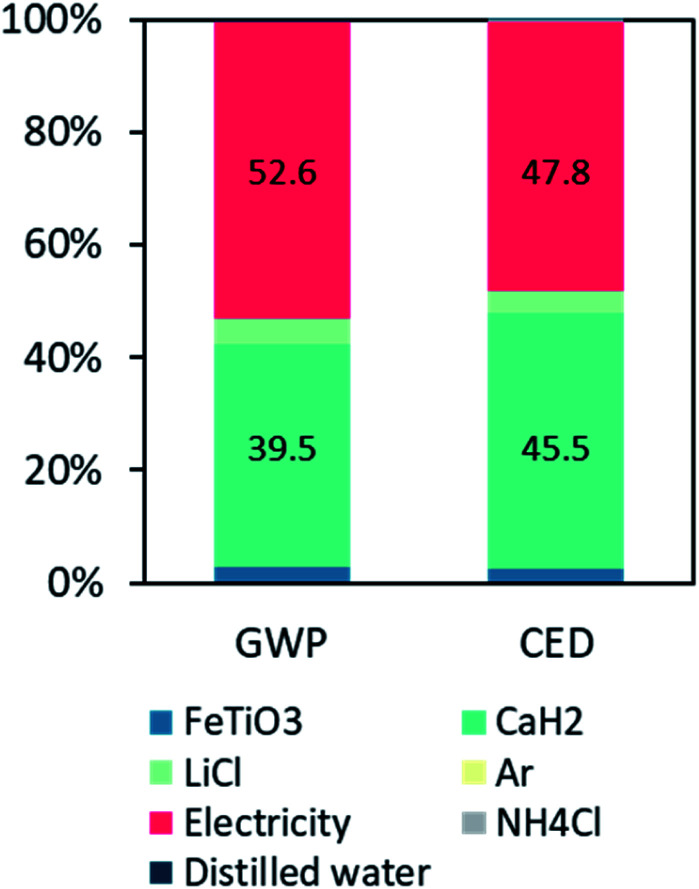
Contributions of the global warming potential (GWP) and cumulative energy demand (CED) of the TiFe nanostructures production.

The LCA results show that to produce 1 kg of TiFe using the FeTiO_3_ method, the associated environmental impacts of global warming potential (GWP) and cumulative energy demand (CED) were 32.93 kg CO_2_e and 527 MJ, respectively. Fig. 7 shows the breakdown of their contributions. For the GWP, the contribution of the furnace electricity consumption accounted for 52%, and the CaH_2_ production accounted for 39%. Also, the LiCl and FeTiO_3_ production accounted for 4% and 2%, respectively. For the CED, similar contributions were observed (47% and 45%) for the electricity and CaH_2_, respectively. The environmental electricity hotspot was calculated based on the average electricity mixed in Japan, which is fossil-fuel intensive, or 0.67 kg of CO_2_e emissions per kW h. This inferred that any innovation to reduce the reaction temperature and duration is critical in reducing the environmental impact. Another important finding was that the bottleneck in this proposed TiFe production method was the use of the CaH_2_ reducing agent. CaH_2_ was produced from a direct combination of calcium and hydrogen. The industrial production of calcium is either through electrolysis or through the metallothermic reduction of calcium chloride,^33^ and both processes are extremely energy-intensive. Until an alternative reducing agent with the same effect is found, this small amount of CaH_2_ would contribute to nearly half the impact.

To the best of our knowledge, this work includes the first LCA study on TiFe production. We wanted to compare our FeTiO_3_ method to other methods in a comprehensive manner, but this was not possible due to the lack of detailed information. However, some clear advantages could be observed. First, for other combustion syntheses^18,19^ that require similar energy-intensive reduction materials (such as Ca and Mg metals), the reaction temperatures are much higher. Second, for other electrochemical methods,^17^ long and continuous electricity supply in addition to higher temperatures is needed. Finally, for the conventional method using pure titanium and iron, the upstream process of obtaining titanium from ilmenite results in up to 30.16 kg of CO_2_e per kg-Ti, which outweighs the impact of the used materials in our method. Furthermore, based on our in-house measurements, the electricity consumption of an arc melting furnace was found to be about five times that of an electric furnace.

## Conclusions

In this study, intermetallic TiFe nanostructures were chemically prepared from titanium sources (TiFeO_3_ and TiO_2_) at as low as 600 °C. The unique layered/spherical morphologies were observed in the obtained samples, and they seemed to be originating from the oxide precursors. The precursor originated morphologies could be allowed by the low reduction temperature, through which the TiFe grain growth rate was very slow. An environmental evaluation of the prospective TiFe production was performed from a product lifecycle perspective to ensure sustainability. The LCA results suggested that our FeTiO_3_ method is likely more environmentally friendly than the existing methods in terms of GWP and CED due to the advantage of the lower temperature and shorter duration.

## Author contributions

YK carried out the experiments including synthesis and characterization. YK also conceptualized the project and supervised the research work. HYT performed the LCA analysis. NH discussed the results, helped to prepare the manuscript.

## Conflicts of interest

There are no conflicts to declare.

## Supplementary Material

NA-003-D1NA00251A-s001
