# Supplementary material for: Chemical synthesis of unique intermetallic TiFe nanostructures originating from the morphology of oxide precursors
Source: Nanoscale Adv. 2021 Jul 13;3(18):5284–91. doi: 10.1039/d1na00251a (PMC9417203; doi:10.1039/d1na00251a)
Supplement: NA-003-D1NA00251A-s001 [file NA-003-D1NA00251A-s001.pdf]

Electronic Supplementary Material (ESI) for Nanoscale Advances.
This journal is © The Royal Society of Chemistry 2021

Electronic Supplementary Information

Chemical Synthesis of Unique Intermetallic TiFe Nanostructures Originating from the Morphology of Oxide Precursors

Yasukazu Kobayashi [*,a], Heng Yi Teah [b], and Nobuko Hanada [c]

a Interdisciplinary Research Center for Catalytic Chemistry, National Institute of Advanced Industrial Science and Technology (AIST), 1-1-1 Higashi, Tsukuba, Ibaraki 305-8565, Japan

E-mail: yasu-kobayashi@aist.go.jp

b Waseda Research Institute for Science and Engineering, Waseda University, Tokyo 169-8555, Japan

c Department of Applied Chemistry, Waseda University, 3-4-1 Okubo, Shinjuku-ku, Tokyo, 169-8555, Japan

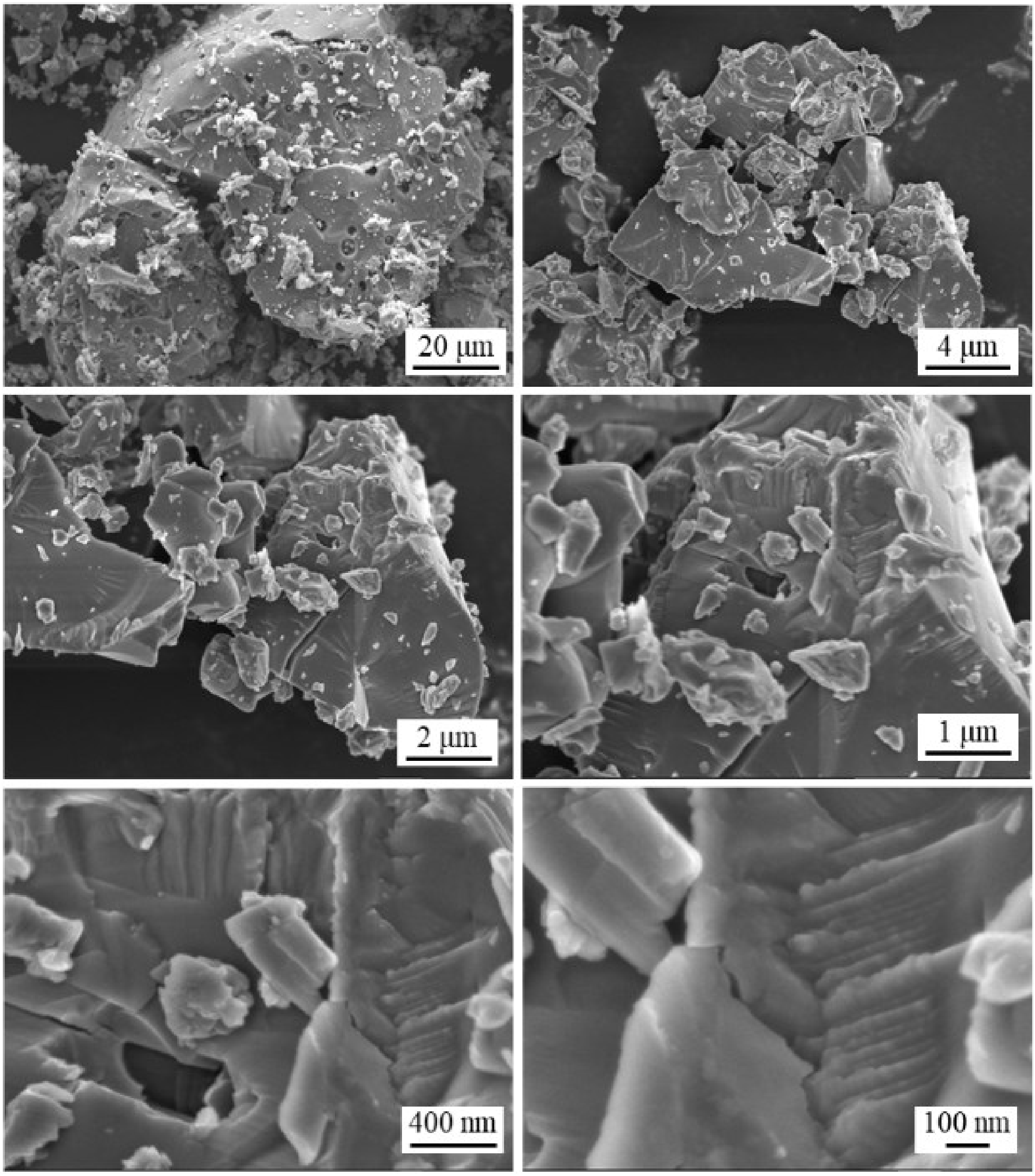

Fig. S1 SEM images for commercial $FeTiO_3$.

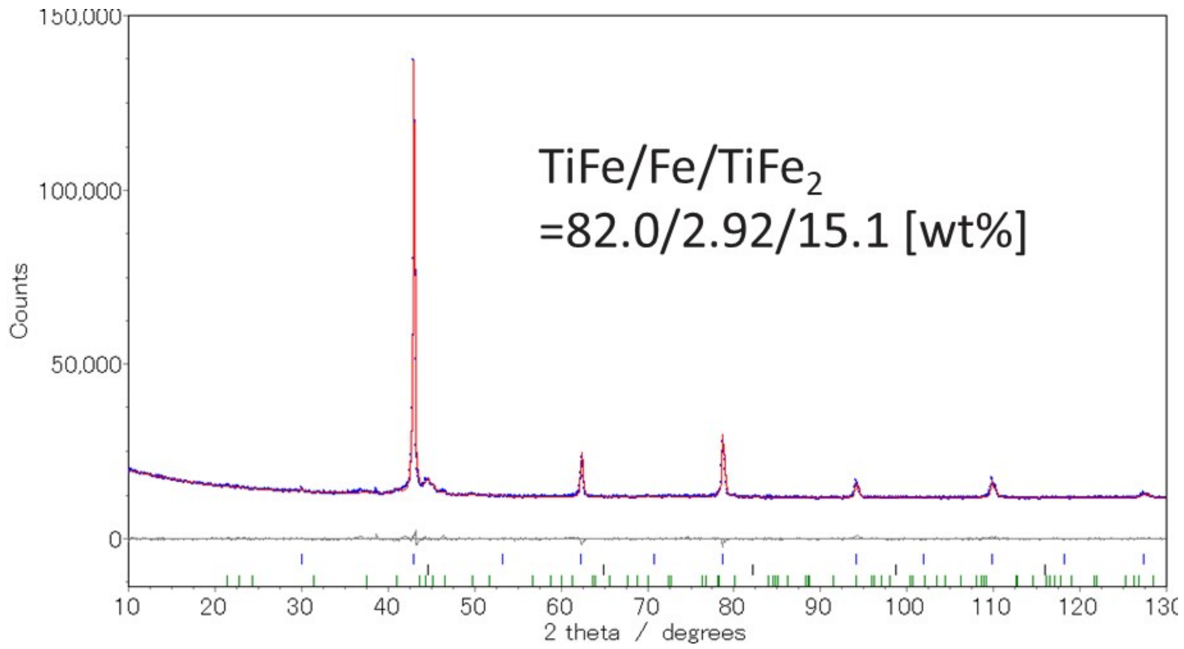

Fig. S2 Rietveld refinement results for TiFe(RDT-FTO). Observed (blue dots), calculated (red line), and difference (gray line) profiles are presented. The vertical bars at the bottom show the calculated positions for TiFe (blue), Fe (black) and $TiFe_2$ (green).

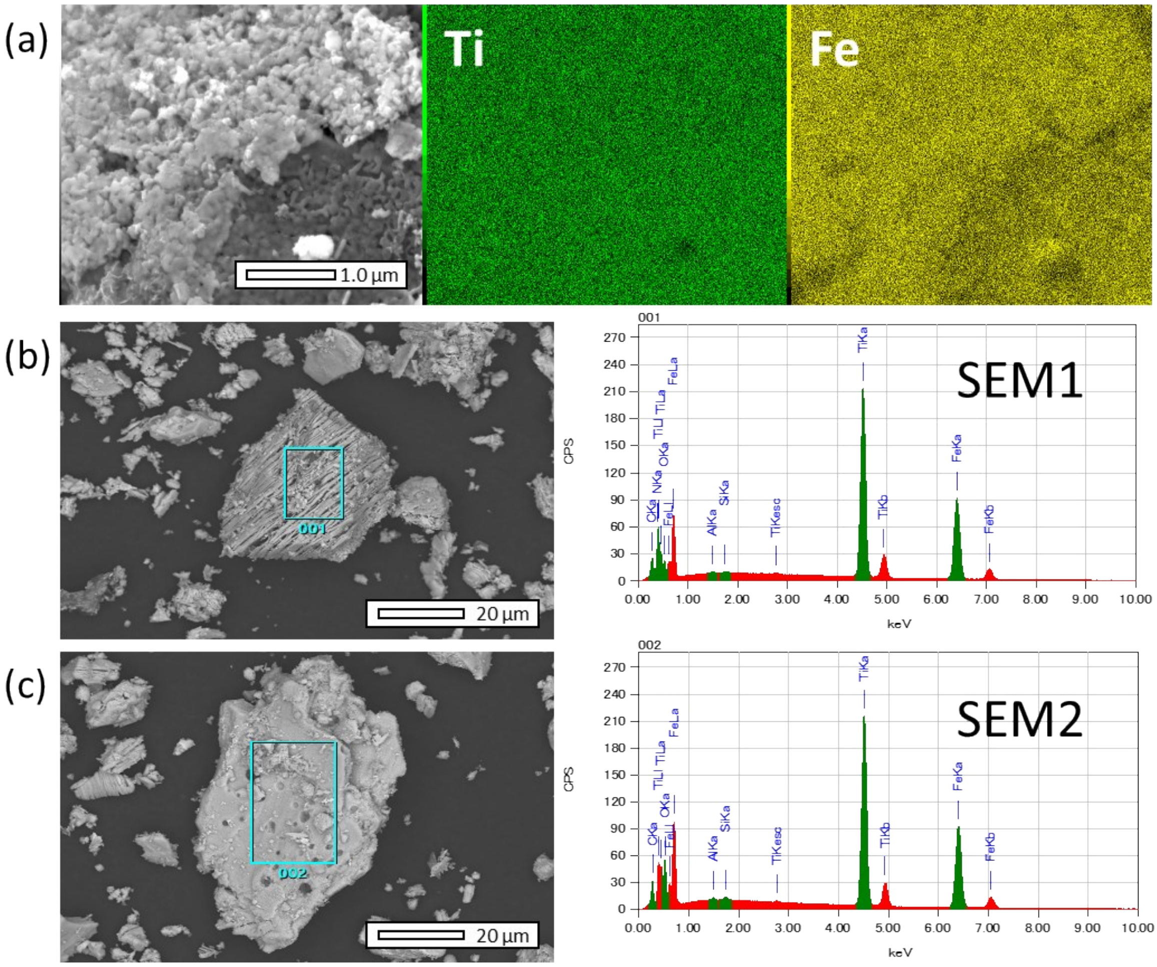

Fig. S3 (a) Elemental mappings and SEM-EDS analysis at two positions of (b) SEM1 and (c) SEM2 for TiFe (RDT-FTO).

Table S1 Molar ratios of detected elements by SEM-EDS for TiFe (RDT-FTO) and TiFe (RDT-TO).

Sample	Molar ratio of detected elements [mol%]						
	Method[a)]	Ti	Fe	O	Al	Si	Ca
TiFe(RDT-FTO)	SEM1	46.34	49.14	4.27	0.17	0.08	-
	SEM2	43.06	45.46	11.07	0.18	0.23	-
TiFe(RDT-TO)	SEM1	43.59	47.52	8.19	0.14	0.21	0.35
	SEM2	43.50	46.40	9.28	0.19	0.33	0.30

a) Element ratios were measured at 2 different positions of SEM1 and SEM2 for both samples.

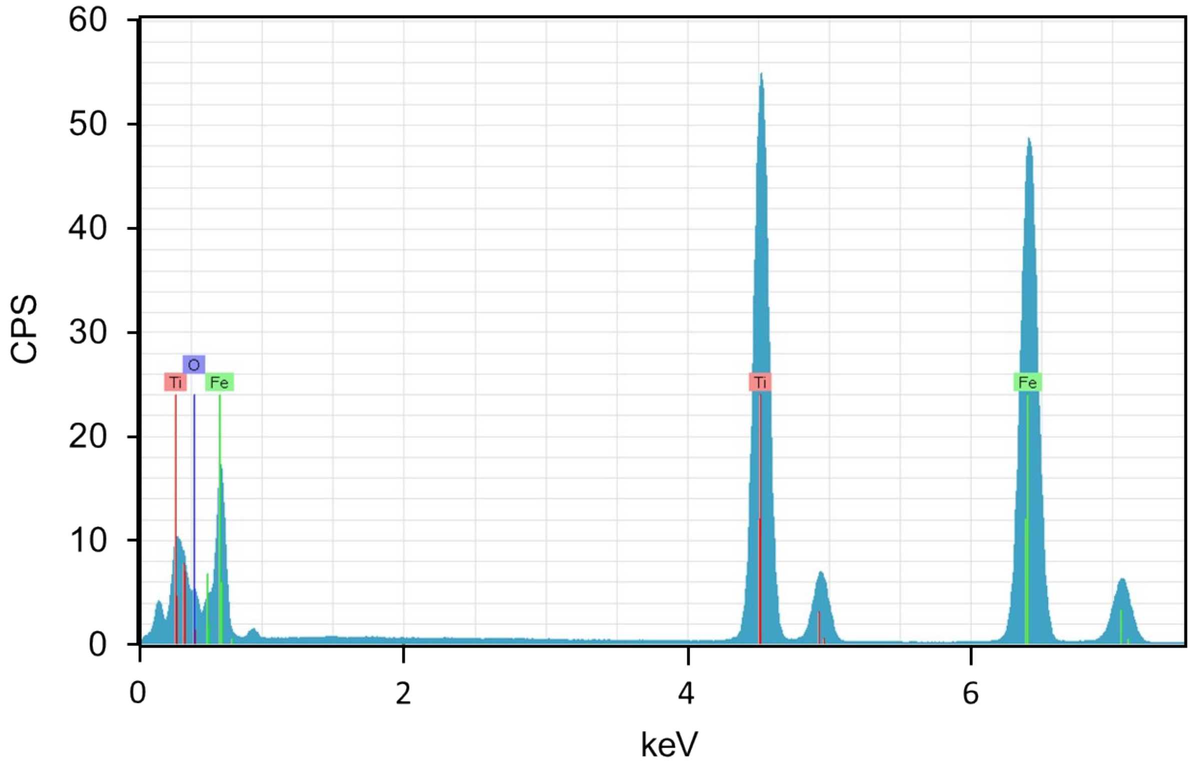

Fig. S4 TEM-EDS spectrum observed at a marked position in Fig. 2(b) for TiFe (RDT-FTO).

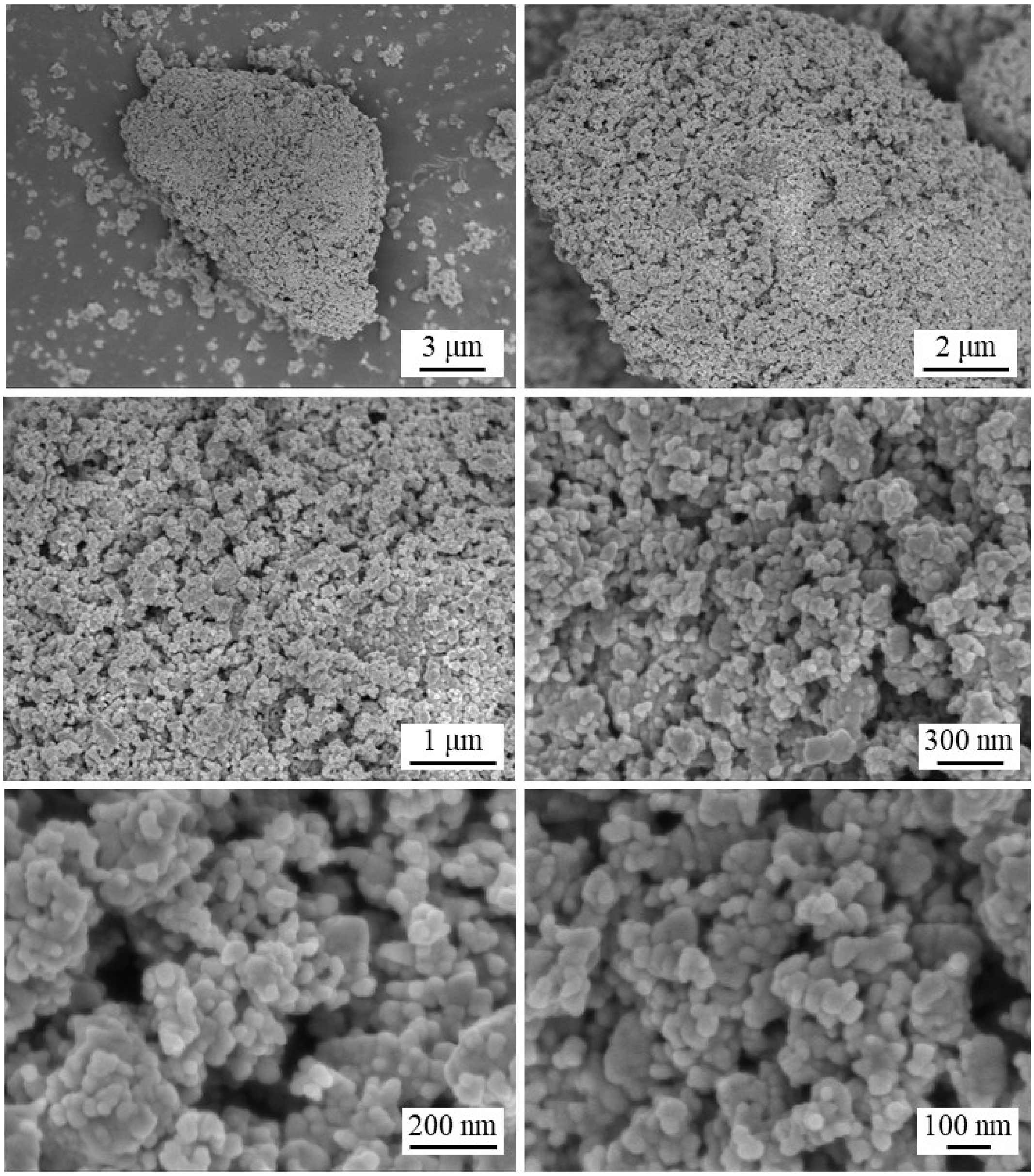

Fig. S5 SEM images for TiFe (Pre-TO).

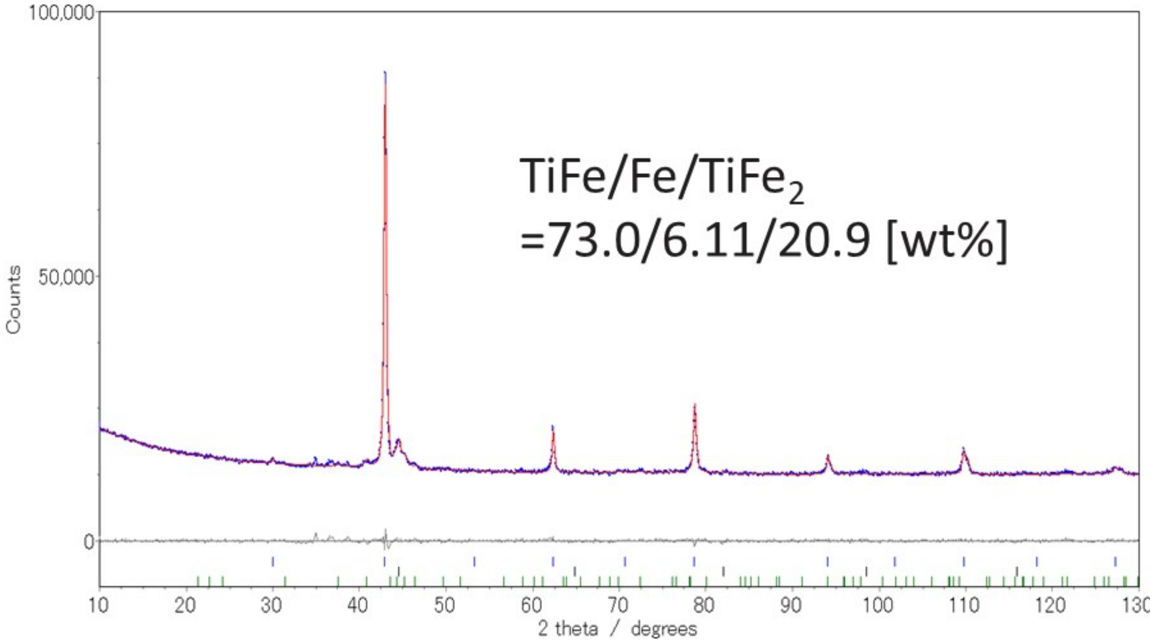

Fig. S6 Rietveld refinement results for TiFe(RDT-TO). Observed (blue dots), calculated (red line), and difference (gray line) profiles are presented. The vertical bars at the bottom show the calculated positions for TiFe (blue), Fe (black) and $TiFe_2$ (green).

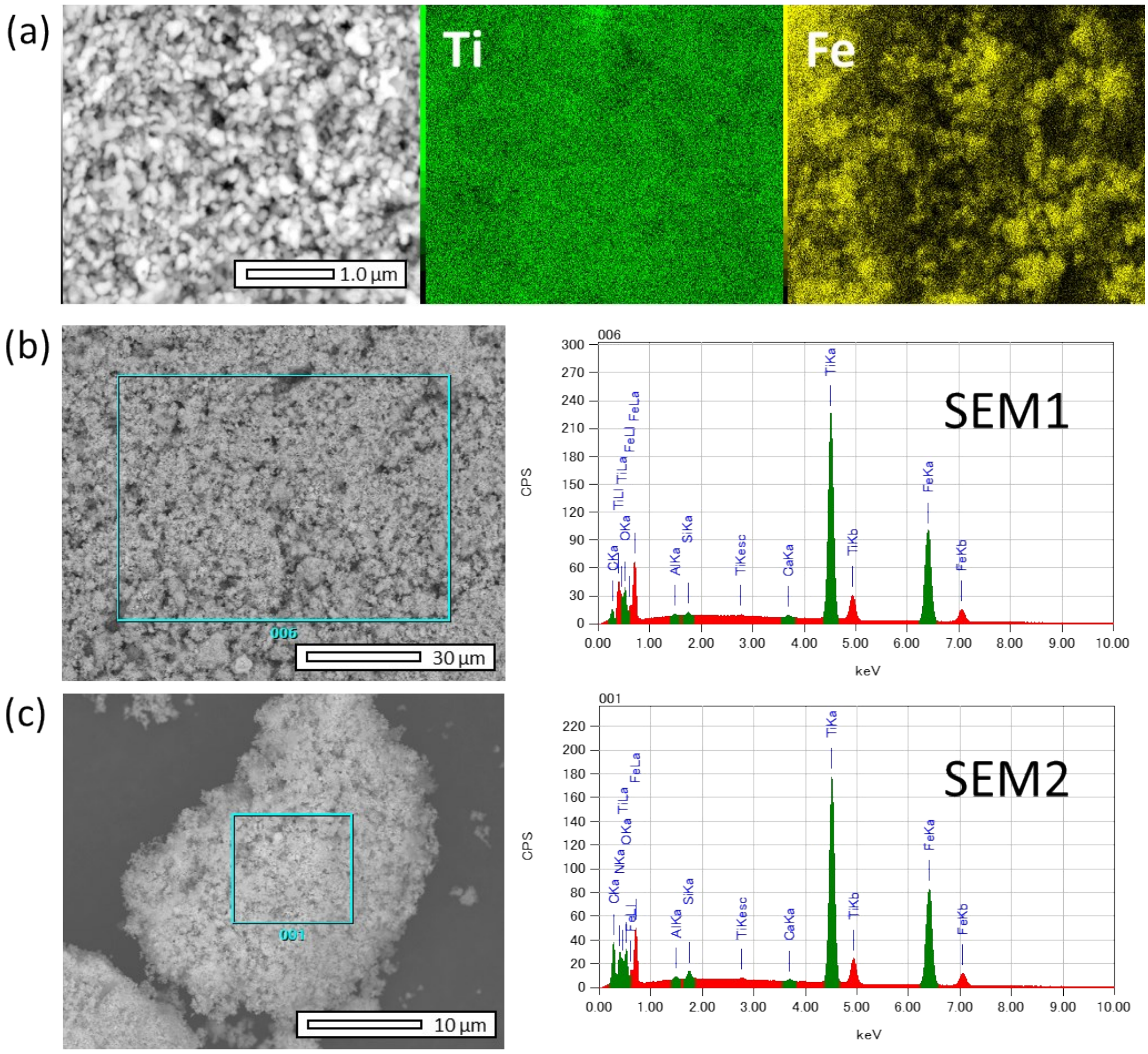

Fig. S7 (a) Elemental mappings and SEM-EDS analysis at two positions of (b) SEM1 and (c) SEM2 for TiFe (RDT-TO).

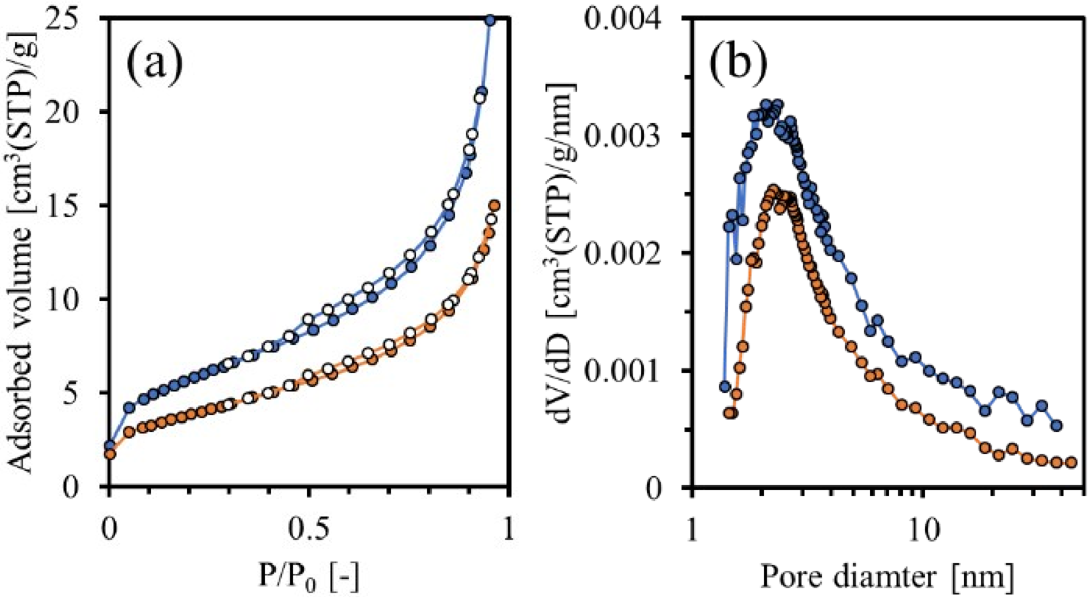

Fig. S8 (a) Adsorption and desorption isotherms of nitrogen and (b) pore size distributions. Orange: TiFe (RDT-FTO), Blue: TiFe (RDT-TO).
